# Radiomic signatures based on pretreatment 18F-FDG PET/CT, combined with clinicopathological characteristics, as early prognostic biomarkers among patients with invasive breast cancer

**DOI:** 10.3389/fonc.2023.1210125

**Published:** 2023-07-27

**Authors:** Tongtong Jia, Qingfu Lv, Xiaowei Cai, Shushan Ge, Shibiao Sang, Bin Zhang, Chunjing Yu, Shengming Deng

**Affiliations:** ^1^ Department of Nuclear Medicine, the First Affiliated Hospital of Soochow University, Suzhou, China; ^2^ Department of General Surgery, The First Affiliated Hospital of Soochow University, Suzhou, China; ^3^ Department of Nuclear Medicine, The Affiliated Suqian First People’s Hospital of Nanjing Medical University, Suqian, China; ^4^ Department of Nuclear Medicine, Affiliated Hospital of Jiangnan University, Wuxi, China

**Keywords:** breast cancer, radiomic, PET/CT, prognosis, nomogram, biomarker

## Abstract

**Purpose:**

The aim of this study was to investigate the predictive role of fluorine-18 fluorodeoxyglucose positron emission tomography/computed tomography (^18^F-FDG PET/CT) in the prognostic risk stratification of patients with invasive breast cancer (IBC). To achieve this, we developed a clinicopathologic-radiomic-based model (C-R model) and established a nomogram that could be utilized in clinical practice.

**Methods:**

We retrospectively enrolled a total of 91 patients who underwent preoperative ^18^F-FDG PET/CT and randomly divided them into training (n=63) and testing cohorts (n=28). Radiomic signatures (RSs) were identified using the least absolute shrinkage and selection operator (LASSO) regression algorithm and used to compute the radiomic score (Rad-score). Patients were assigned to high- and low-risk groups based on the optimal cut-off value of the receiver operating characteristic (ROC) curve analysis for both Rad-score and clinicopathological risk factors. Univariate and multivariate Cox regression analyses were performed to determine the association between these variables and progression-free survival (PFS) or overall survival (OS). We then plotted a nomogram integrating all these factors to validate the predictive performance of survival status.

**Results:**

The Rad-score, age, clinical M stage, and minimum standardized uptake value (SUV_min_) were identified as independent prognostic factors for predicting PFS, while only Rad-score, age, and clinical M stage were found to be prognostic factors for OS in the training cohort. In the testing cohort, the C-R model showed superior performance compared to single clinical or radiomic models. The concordance index (C-index) values for the C-R model, clinical model, and radiomic model were 0.816, 0.772, and 0.647 for predicting PFS, and 0.882, 0.824, and 0.754 for OS, respectively. Furthermore, decision curve analysis (DCA) and calibration curves demonstrated that the C-R model had a good ability for both clinical net benefit and application.

**Conclusion:**

The combination of clinicopathological risks and baseline PET/CT-derived Rad-score could be used to evaluate the prognosis in patients with IBC. The predictive nomogram based on the C-R model further enhanced individualized estimation and allowed for more accurate prediction of patient outcomes.

## Introduction

Breast cancer (BC) is now the leading cause of malignancy incidence and tumor-related deaths among females worldwide, surpassing lung cancer (1). The comprehensive therapy of BC, including surgery, chemotherapy, radiotherapy, and targeted treatments, has been effective in reducing locoregional or distant recurrences and prolonging survival (2–4). However, the intrinsic intratumoral heterogeneity of BC has resulted in distinct patterns of tumor progression, metastasis formation, and therapy resistance (5). Despite active therapies, some patients still develop various forms of resistance, which has not altered mortality outcomes (6, 7). Clinicians have made initial prognostic predictions and individualized therapies based on the tumor-node-metastasis (TNM) staging system and molecular classification (8, 9). However, it remains difficult to precisely predict the prognosis of patients with advanced and inoperable BC. This can result in overtreatment or undertreatment due to the high heterogeneity of BC and the complexity of treatment strategies (10).

To improve risk stratification and monitor therapeutic efficacy in BC patients, it is crucial to develop robust image-driven biomarkers. Recently, fluorine-18 fluorodeoxyglucose positron emission tomography/computed tomography (^18^F-FDG PET/CT) has become a common diagnostic tool for BC patients, as it combines functional metabolic quantification with morphological imaging. This technique is useful for initial staging, prognostic assessments, and response monitoring (11, 12). Certain studies have indicated that preoperative metabolic parameters, such as standardized uptake values (SUVs), metabolic tumor volume (MTV), and total lesion glycolysis (TLG), serve as reliable biomarkers associated with the prognosis of BC (13, 14). However, these traditional metabolic factors may not fully capture the spatial distribution between pairs of voxels (15, 16).

Radiomics, which extracts advanced texture features from medical images to non-invasively characterize tumor heterogeneity and predict prognostic response, has emerged as a promising research topic in BC (17–19). However, few studies have investigated the predictive value of baseline PET/CT radiomics in BC prognosis (20, 21). Moreover, combining clinicopathological characteristics with radiomic biomarkers to create predictive signatures and constructing a nomogram is a prevalent and effective approach for achieving prognosis prediction and individualized management (22, 23). In the present study, we aimed to develop a predictive nomogram using the C-R model that combined clinicopathological and radiomic signatures (RSs) based on pretreatment PET/CT to estimate the survival prognosis of BC patients.

## Materials and methods

### Patients and follow-up

This retrospective study was approved by the medical ethics committee of the First Affiliated Hospital of Soochow University and waived additional informed consent (Trial registration number: ChiCTR2300070309). The study was conducted in compliance with the Declaration of Helsinki, and no personal information was disclosed. The total cohort of consecutive patients who were initially diagnosed with BC using ^18^F-FDG PET/CT and confirmed by pathology in our institution between September 2016 and April 2022 were further checked by the following criteria. Inclusion criteria were as follows: a) patients who did not receive any therapy prior to the standard examination of ^18^F-FDG PET/CT; b) patients ultimately diagnosed with invasive carcinoma of BC, including invasive ductal, lobular, or papillary carcinomas, and confirmed by puncture biopsy or surgical specimen; c) patients with available clinical records and pathological data; and d) patients with immunochemistry (IHC) examination, including estrogen receptor (ER), progesterone receptor (PR), human epidermal growth factor receptor 2 (HER2), and Ki-67. Exclusion criteria were as follows: a) patients with the suboptimal quality of ^18^F-FDG PET/CT images due to motion artifacts or abnormal biodistribution of tracer; b) primary lesions with a too small size to be outlined the volume of interest (VOI) for measurement (short-axis diameter <1 cm); c) patients with other types of tumors; d) patients confirmed to have other specific histological types of BC (sarcomas, lymphomas and so on); and e) newly diagnosed patients with a follow-up time of less than 8 months. The enrolled patients were randomly divided into training and validation cohorts at a 7:3 ratio using computer-generated random numbers.

All patients were followed up from the confirmed time of primary diagnosis until the cut-off date of December 30, 2022, using outpatient review data and telephone follow-ups. Progression-free survival (PFS) was defined as the interval between the date of first diagnosis and the first relapse, tumor progression, death, or the last follow-up. Overall survival (OS) was defined as the interval between the date of first diagnosis and death from any cause or the last follow-up. The study endpoints were PFS and OS.

### Image acquisition and reconstruction

According to guidelines from the European Association of Nuclear Medicine (EANM), patients must fast for at least 4 h and ensure that their plasma glucose level is lower than 11.0 mmol/L (about 200 mg/dL) prior to undergoing the 18F-FDG PET/CT procedure in clinical studies (24). Approximately 40-60 min after intravenous injection of ^18^F-FDG (4.07-5.55 MBq/kg), patients were scanned using an integrated PET/CT scanner (Discovery STE, General Electric Medical Systems, Milwaukee, WI, USA) to acquire images from the base of the skull to the midthigh. Low-dose (140 kV, 120 mA) CT images were used for subsequent attenuation correction and anatomic localization of PET images, with acquisition parameters including a transaxial field of view of 70 cm, pitch of 1.75, rotation time of 0.8 s, and slice thickness of 3.75 mm. PET image acquisition was performed at 2-3 min per bed position, with a total of 8-10 bed positions. During image reconstruction, the ordered subset expectation maximization algorithm was used (two iterations and eight subsets) to ensure that reconstructed voxel sizes were within 3.0-4.0 mm in any direction.

### Clinicopathological evaluation

The study collected several clinical factors, including age, menopausal status, tumor location, initial TNM stage, treatment strategies, and diagnosis time. Specimens obtained from core needle biopsy and excisional biopsy were fixed in formalin solution, embedded in paraffin, and stained with hematoxylin and eosin (H&E staining). Stained sections were evaluated by independently two experienced pathologists to confirm the histopathological type. The expressions of ER, PR, HER2, and Ki-67 were detected using IHC. ER and PR were considered positive if there was a proportion of at least 1% of nuclear staining. HER2 status was confirmed using a combination of IHC scores and fluorescence *in situ* hybridization (FISH), where a positive result was defined as IHC 2+ and FISH gene amplification or IHC 3+ (25). Ki-67 cell nuclear staining of ≥ 30% was considered a high expression.

### VOI segmentation and radiomic feature extraction

The Local Image Features Extraction (LifeX) package (version 7.0.0, available at https://www.lifexsoft.org/) was used to automatically match and fuse PET and CT images in DICOM format for quantitative PET/CT analysis (26). Two experienced nuclear medicine physicians, who were blinded to the clinicopathological results, manually segmented the transaxial VOI layer by layer. The VOI was defined by integrating abnormal uptake of ^18^F-FDG on PET and abnormal density on CT, which was optimized by setting a threshold of 40% of the SUVmax to ensure reproducibility (16). Once matched, the RSs (four or six conventional features, nine first-order features, and 32 high-order features) of the CT or PET images could be automatically extracted from the same VOI. To avoid overfitting, significant RSs of the training cohort were selected using correlation analysis, least absolute shrinkage and selection operator (LASSO) regression algorithm, and univariable Cox analysis before model construction (17, 27). Finally, 10-fold cross-validation was used to ensure the robustness of the optimal features.

### Model construction and validation

The Rad-score was calculated using a linear fitting formula, which involved multiplying the remaining features with their respective weighted coefficients to create a radiomic model. Based on the optimal threshold value of the Rad-score, as determined through receiver operating characteristic (ROC) curve analysis, the cohorts were divided into high-risk and low-risk groups. In addition to clinicopathological factors, the Rad-score was further evaluated using Kaplan-Meier (KM) analysis and log-rank tests. All significant factors were entered into a multivariable Cox proportional hazards regression to identify the final subset of prognostic factors. Finally, the radiomic and clinical nomograms were evaluated in the training cohort and then validated in the testing cohort. To evaluate the discriminative ability, calibration, and clinical usefulness of the models, we employed the Harrell concordance index, calibration curves, and decision curve analysis (DCA), respectively (28).

### Statistical analysis

**Figure 1:**
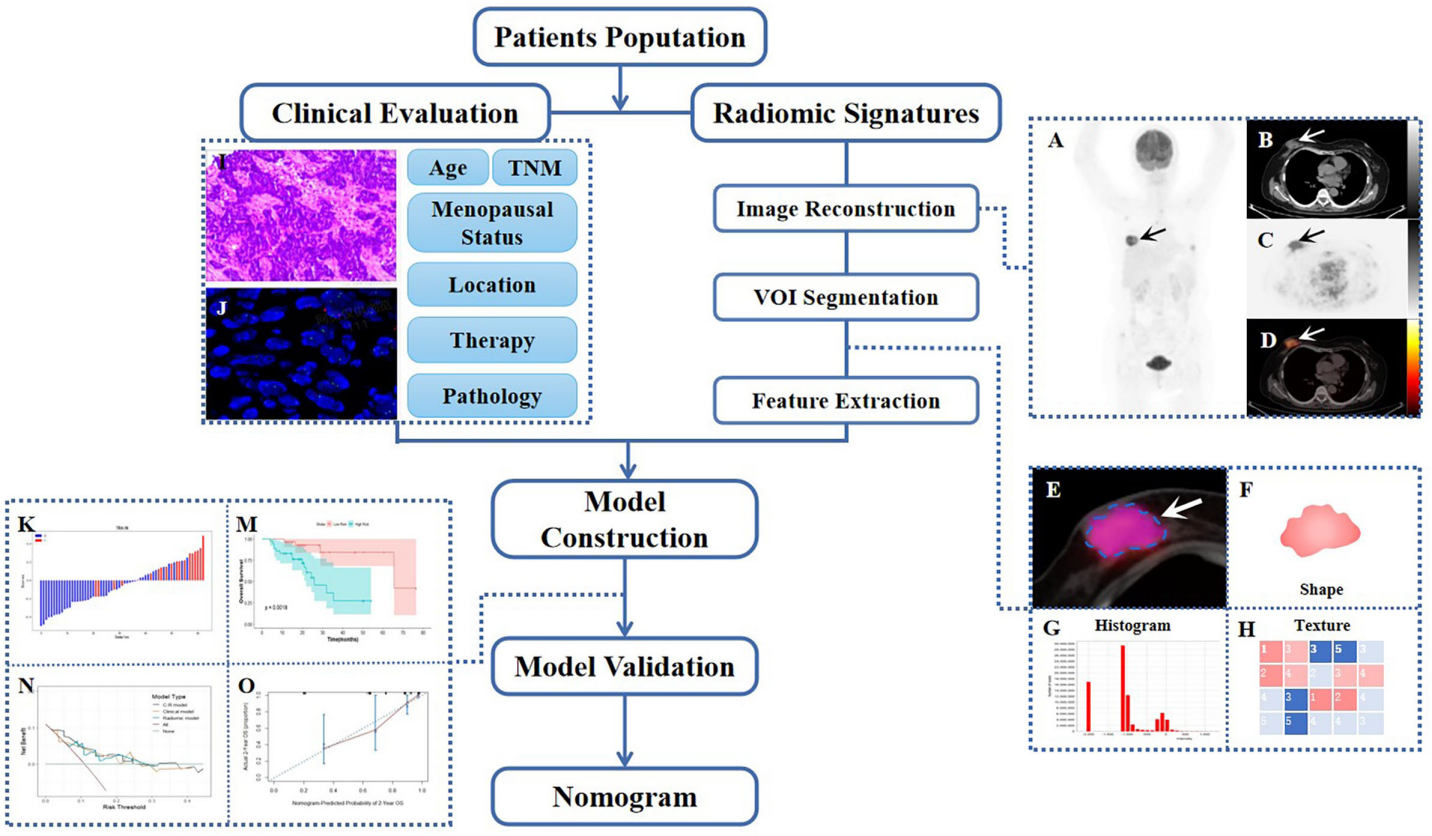
Workflow diagram of this retrospective study. The typical example of PET/CT image reconstruction in a patient with BC, whole-body maximum intensity projection (MIP) of PET image **(A)**, axial views of low-dose CT, PET or PET/CT infusion scans **(B–D)**. Arrows point to tumor lesions. The dotted line circles the VOI in magnified PET/CT image **(E)**, from which first-order **(F, G)** and high-order **(H)** radiomic features were extracted. The IHC and FISH of representatively pathological section **(I, J)**. Waterfall plot of radiomic score (Rad-score) and KM curve of survival analysis during model construction **(K, M)**, DCA, and calibration curve during model validation **(N, O)**.

Statistical data were calculated and analyzed using IBM SPSS Statistics (version 26.0, IBM Corp), Python (version 3.0, https://www.python.org), MedCalc software (MedCalc Software, Ostend, Belgium), and R (version 4.2.1, http://www.R-project.org). The normality and homogeneity of variance for continuous data were evaluated using the Kolmogorov-Smirnov test and Levene’s test, respectively. The independent t-test and Mann-Whitney U test were then used to evaluate any differences in baseline characteristics between the training and testing sets. Meanwhile, the Chi-square test and Fisher’s exact test were applied to analyze categorical variables. For the final survival analysis, quantitative variables were transformed into dichotomous variables to conduct further univariate and multivariate Cox analyses, as well as to estimate hazard ratios (HRs). A two-sided p-value of less than 0.05 was considered statistically significant. **Figure 1** provides an overview of the study’s workflow.

## Results

### Patient population

**Figure 2:**
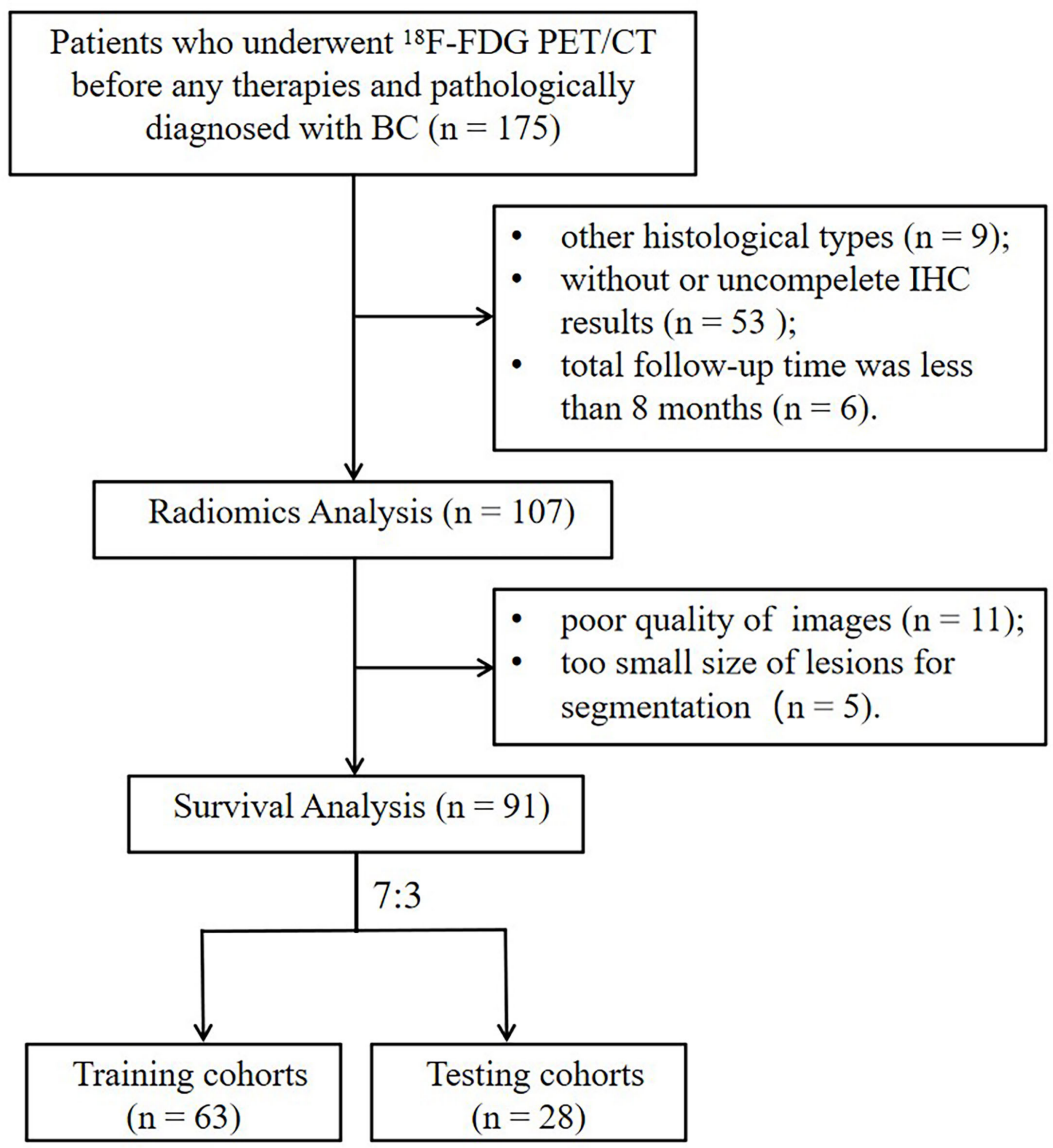
The flowchart of the selection process according to eligibility and exclusive criteria. BC, breast cancer; IHC, immunohistochemistry.

In this retrospective study, 91 patients with BC pretreatment who met the inclusion criteria were selected (in **Figure 2**). **Table 1** summarizes the clinical characteristics of the training and validation cohorts, and there were no statistically significant biases in patient distribution between the two cohorts (all p > 0.05). During the follow-up period from the time of primary diagnosis (median: 20 months, range: 4 - 95 months), 42 out of 91 patients (46.2%) developed PFS endpoints, and 27 out of 91 patients (29.7%) died. Patients without any PFS events who survived at least 8 months after cancer diagnosis were considered as the control group (n = 49). The event rates of PFS (46.0% and 46.4%, respectively) and OS (30.2% and 28.6%, respectively) were not significantly different between the two cohorts, indicating a balanced distribution.

**Table 1:** The clinicopathological features of the enrolled population.

	Total (n=91)	Training (n=63)	Testing (n=28)	t/x^2^	p
Age (years)	53.33 ± 13.46	53.05 ± 13.47	54.00 ± 13.66	-0.307	0.760
Menopausal Status				0.266	0.606
Premenopausal	34 (37.4%)	25 (39.1%)	9 (33.3%)		
Postmenopausal	57 (62.6%)	39 (60.9%)	18 (66.7%)		
Tumor Location				3.650	0.056
Left	51 (56.0%)	40 (62.5%)	11 (40.7%)		
Right	40 (44.0%)	24 (37.5%)	16 (59.3%)		
Histological Type				0.809	0.368
IDC	82 (90.1%)	56 (87.5%)	26 (96.3%)		
*Other	9 (9.9%)	8 (12.5%)	1 (3.7%)		
Clinical Stage				1.866	0.393
I-II	22 (24.2%)	18 (28.1%)	4 (14.8%)		
III	34 (37.4%)	23 (35.9%)	11 (40.7%)		
IV	35 (38.4%)	23 (35.9%)	12 (44.4%)		
Clinical T Stage				0.646	0.886
cT1	15 (16.5%)	11(17.2%)	4 (14.8%)		
cT2	38 (41.8%)	28 (43.8%)	10 (37.0%)		
cT3	3 (3.3%)	2 (3.1%)	1 (3.7%)		
cT4	35 (38.4%)	23 (35.9%)	12 (44.4%)		
Clinical N Stage				2.949	0.223
cN0	16 (17.6%)	14 (21.9%)	2 (7.4%)		
cN1-2	39 (42.8%)	25 (39.1%)	14 (51.9%)		
cN3	36 (39.6%)	25 (39.1%)	11 (40.7%)		
Clinical M Stage				0.187	0.665
cM0	57 (62.6%)	41 (64.1%)	16 (59.3%)		
cM1	34 (37.4%)	23 (35.9%)	11 (40.7%)		
ER Status				0.033	0.856
Positive	56 (61.5%)	39 (60.9%)	17 (63.0%)		
Negative	35 (38.5%)	25 (39.1%)	10 (37.0%)		
PR Status				0.892	0.345
Positive	37 (40.7%)	24 (37.5%)	13 (48.1%)		
Negative	54 (59.3%)	40 (62.5%)	14 (51.9%)		
HER2 Status				1.133	0.287
Positive	31 (34.1%)	24 (37.5%)	7 (25.9%)		
Negative	60 (65.9%)	40 (62.5%)	20 (74.1%)		
Molecular Subtype				0.707	0.892
HR+/HER2-	43 (47.2%)	29 (45.3%)	14 (51.9%)		
HR+/HER2+	14 (15.4%)	10 (15.6%)	4 (14.8%)		
HER2+	18 (19.8%)	14 (21.9%)	4 (14.8%)		
TNBC	16 (17.6%)	11 (17.2%)	5 (18.5%)		
Ki-67				0.193	0.66
<30%	30 (33.0%)	22 (34.4%)	8 (29.6%)		
≥30%	61 (67.0%)	42 (65.6%)	19 (70.4%)		
Treatment				1.059	0.589
NAC	21 (23.0%)	13 (20.3%)	8 (29.6%)		
PCT	27 (29.7%)	19 (29.7%)	8 (29.6%)		
Other	43 (47.3%)	32 (50.0%)	11 (40.7%)		

Descriptive statistics were summarized with mean±standard deviation and analyzed by independent t-test. Categorical variables were shown as numbers and percentages and analyzed by Pearson's Chi-square test or Fisher's exact test. *Other: including invasive lobular or papillary carcinomas. Abbreviations: IDC, invasive ductal carcinoma; ER, estrogen receptor; PR, progesterone receptor; HER2, human epidermal growth factor receptor 2; TNBC, triple-negative breast cancer; NAC, neoadjuvant chemotherapy; PCT, postoperative chemotherapy.

### RS selection and Rad-score construction

**Figure 3:**
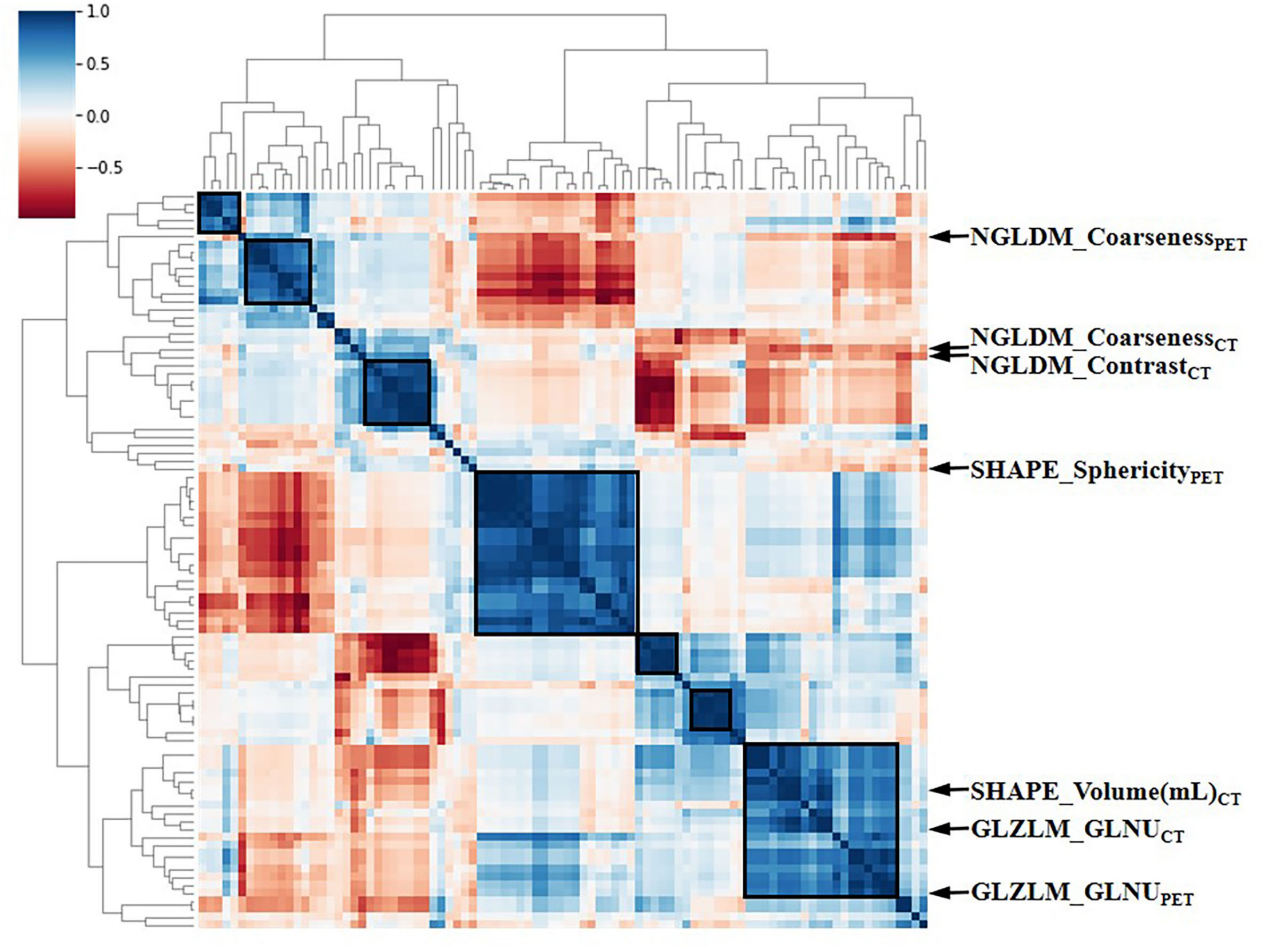
The heat map of Pearson correlation analysis among 97 radiomic features. Correlation coefficients were displayed by color scale. Boxes circled the clusters that were closely related. Representative RSs were marked by arrows. RSs, radiomic signatures; NGLDM, Neighboring Gray-level dependence matrix; GLZLM, Gray-Level Zone Length Matrix; GLNU, Gray-Level Non-Uniformity for zone.

**Figure 4:**
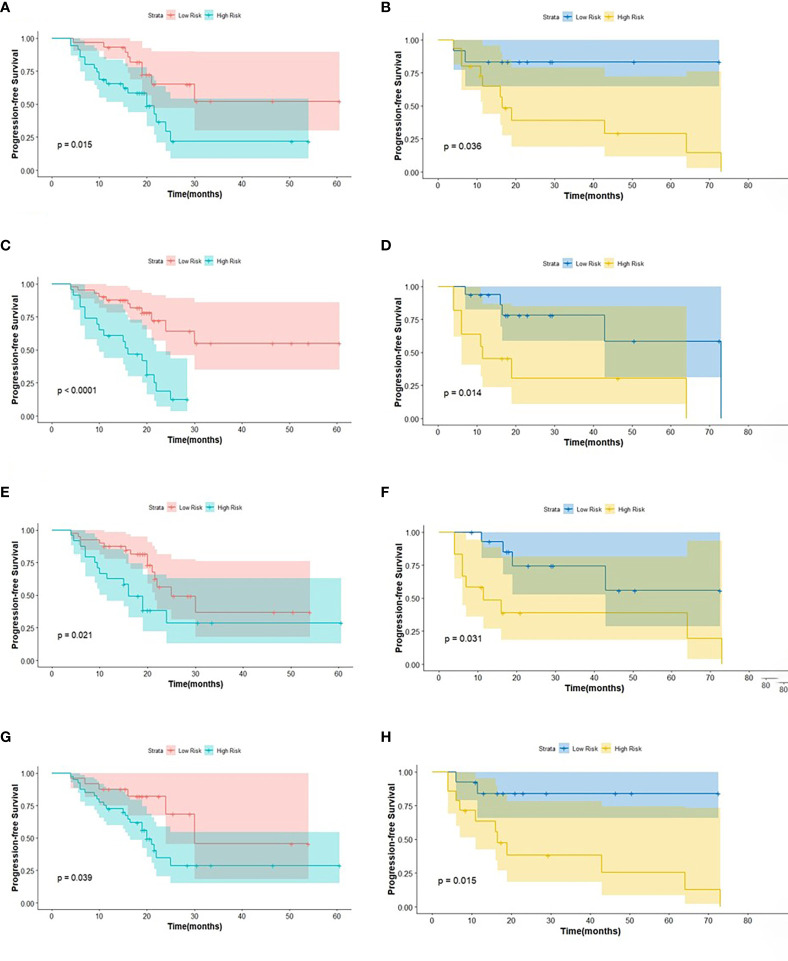
The KM analysis of independent factors for predicting PFS in the training cohort and in the testing cohort (**A, B**: age; **C, D**: clinical M stage; **E, F**: SUV_min_; **G, H**: Rad-score).

**Figure 5:**
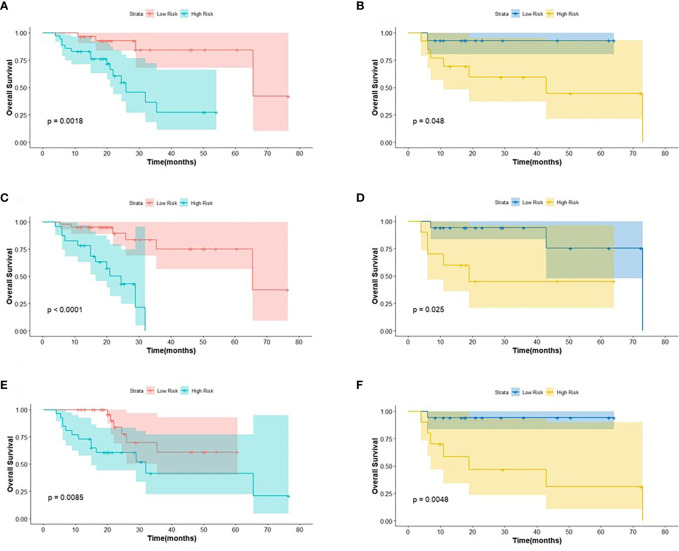
The KM analysis of meaningful biomarkers for predicting OS in the training cohort and in the testing cohort (**A, B**: age; **C, D**: clinical M stage; **E, F**: Rad-score).

The intra-class correlation coefficient (ICC) of the extracted radiomic features was above 0.75 between the two experienced nuclear medicine physicians, and they reached a final agreement in consensus. Pearson correlation analysis between the RSs was visualized in **Figure 3**, and several strongly correlated clusters were circled by black boxes. After using LASSO for dimensionality reduction to remove redundant features (with zero coefficients), the most significant prognostic signatures were selected to calculate the Rad-score in the training cohort. Finally, a total of four RSs consisting of two CT RSs [SHAPE_Volume(mL)_CT_ and GLZLM_GLNU_CT_] and two PET RSs (NGLDM_Coarseness_PET_ and GLZLM_GLNU_PET_) were chosen for predicting PFS. With regard to OS, two CT RSs (NGLDM_Coarseness_CT_ and NGLDM_Contrast_CT_) and three PET RSs (SHAPE_Sphericity_PET_, NGLDM_Coarseness_PET_, and GLZLM_GLNU_PET_) were involved in the predictive formula as follows: Rad-score_PFS_= - 0.0941130 × SHAPE_Volume(mL)_CT_- 0.033147 × GLZLM_GLNU_CT_ + 0.057186 × NGLDM_Coarseness_PET_- 0.105334 × GLZLM_GLNU_PET_. Rad-score_OS_ = 0.106539 × NGLDM_Coarseness_CT_ + 0.050655×NGLDM_Contrast_CT_ + 0.049265×SHAPE_Sphericity_PET_ + 0.097559×NGLDM_Coarseness_PET_ - 0.013341×GLZLM_GLNU_PET_. The median Rad-score calculated using the above formula was 0.2923 (range: 0.0475 - 0.5508) for PFS and 0.4227 (range: 0.1636 - 0.9299) for OS. Moreover, the optimum threshold generated from the ROC analysis of PFS and OS was 0.3776 and 0.3197 in the training set, respectively. **Table 2** shows that the area under the curve (AUC) was 0.670 (95% CI: 0.541 - 0.782) for PFS and 0.706 (95% CI: 0.579 - 0.814) for OS. Accordingly, patients were classified into low-score and high-score groups (Rad-score_PFS_> 0.3776; Rad-score_OS_> 0.3197). Then, Rad-score was incorporated into the subsequent survival analysis as a potential prognostic biomarker. Univariate Cox regression indicated that Rad-score was closely associated with both PFS (p=0.039) and OS (p=0.0085) and was shown by KM survival curves (**Figures 4G**, **5E**).

**Table 2:** The Harrell's C-index and AUC results in the training and validation cohorts.

	Training cohort		Validation cohort	
PFS	C-index (95% CI)	AUC (95% CI)	C-index (95% CI)	AUC (95% CI)
Clinical Model	0.761 (0.666 - 0.857)	0.739 (0.614 - 0.841)	0.772 (0.656 - 0.888)	0.816 (0.620 - 0.938)
Radiomic Model	0.613 (0.492 - 0.735)	0.670 (0.541 - 0.782)	0.674 (0.532 - 0.815)	0.599 (0.394 - 0.781)
C-R model	0.786 (0.697 - 0.875)	0.787 (0.667 - 0.880)	0.816 (0.685 - 0.947)	0.830 (0.636 - 0.946)
OS				
Clinical model	0.794 (0.703 - 0.885)	0.731 (0.605 - 0.834)	0.824 (0.666-0.981)	0.780 (0.579 - 0.915)
Radiomic model	0.730 (0.640 - 0.819)	0.706 (0.579 - 0.814)	0.754 (0.584-0.923)	0.711 (0.505 - 0.867)
C-R model	0.878 (0.816 - 0.940)	0.789 (0.669 - 0.881)	0.882 (0.781-0.984)	0.859 (0.671 - 0.962)

C-index, concordance index; CI, confidence interval; AUC, area under the curve; C-R model, clinicopathologic-radiomic-based model.

### Combined model construction

**Figure 6:**
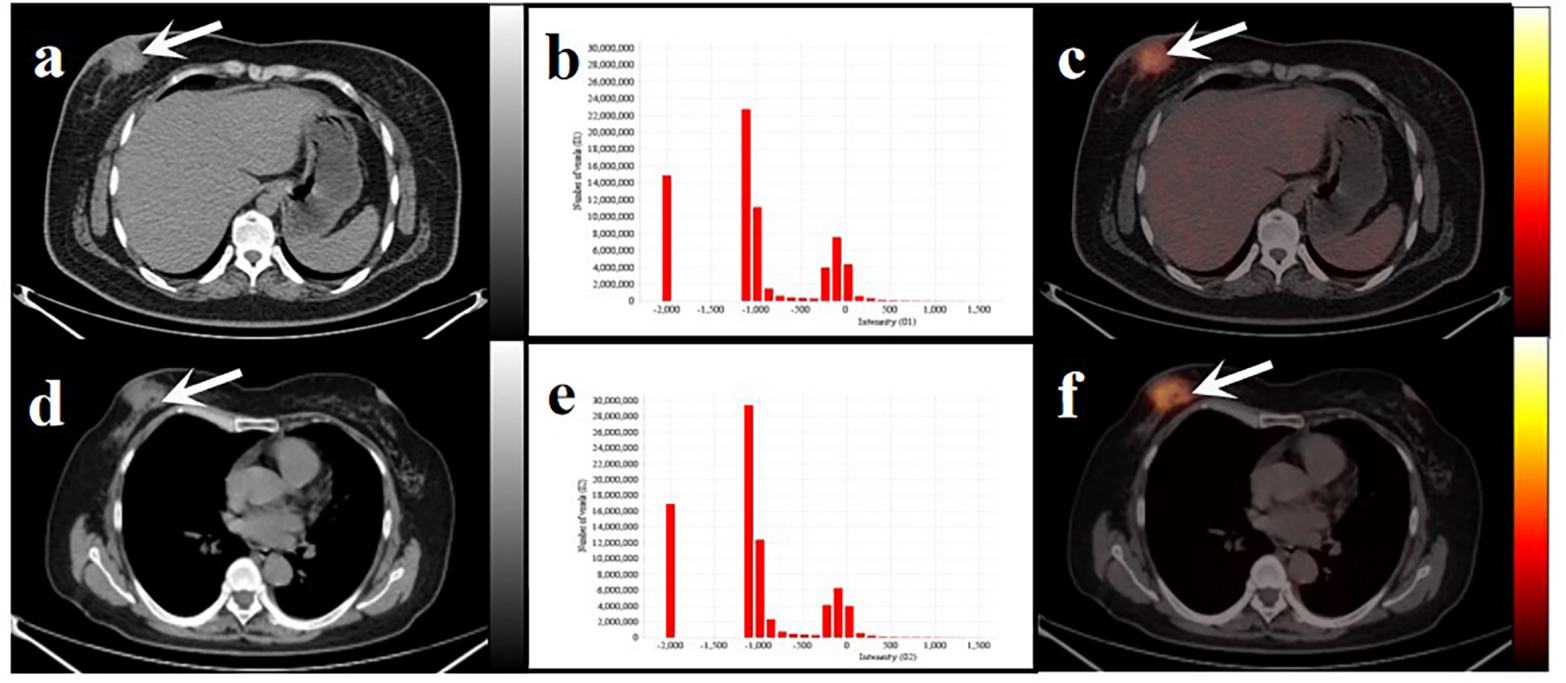
Two women with similar lesions were initially diagnosed with BC. Axial low-dose CT images **(A, D)** and infusion PET/CT images **(C, F)**. Black and white arrows point to the primary lesions located on the right breast. The histograms of CT intensity for the VOI **(B, E)**. The former (age: 32 years, IDC: grade 2, molecular type: HR+/HER2-, clinical stage: T4N1M0, Rad-score: 0.1647) who underwent neoadjuvant chemotherapy didn’t have any obvious disease progression during the 19-month follow-up. The latter (age:61 years, IDC: grade 2, molecular type: TNBC, clinical stage: T4N3M1, Rad-score: 0.3089) underwent postoperative chemotherapy and died after 20 months. These primary lesions were similar on PET/CT images but showed significant differences in the histograms of the radiomic features and clinical outcomes.

The log-rank test was used to perform a univariate analysis of clinicopathological and radiomic features in predicting the prognosis of BC. Additionally, characteristics with a p-value <0.05 were included in the final multivariate Cox regression (**Table 3**). In the univariate analysis of PFS, it was found that age, menopausal status, clinical stage, clinical N stage, clinical M stage, ER status, PR status, Ki-67, SUV parameters (SUV_max_, SUV_min_, SUV_mean_, and SUV_peak_), and Rad-score were potential biomarkers. Among them, age (HR = 3.532, P = 0.013), clinical M stage (HR = 2.977, P = 0.017), SUV_min_ (HR = 4.240, P = 0.001), and Rad-score (HR = 2.660, P = 0.044) were independent factors for prognosis in the multivariate proportional hazards model (**Figure 6**). Meanwhile, age (HR = 5.644, P = 0.013), clinical M stage (HR = 3.499, P = 0.057), and Rad-score (HR = 3.627, P = 0.026) were selected from significant factors (age, menopausal status, clinical stage, clinical N stage, clinical M stage, PR status, and Rad-score) to construct the integrated model for predicting OS. Furthermore, the remaining factors had a strong predictive value for PFS and OS in the training cohort, which was similar to the results observed in the validation cohort through KM analyses (**Figures 4**, **5**).

**Table 3:** The results of the univariate and multivariate Cox regression analysis.

Variable	PFS				OS			
	Log Rank		Cox Regression		Log Rank		Cox Regression	
	x2	p	HR (95%CI)	p	x2	p	HR (95%CI)	p
Age (years)	5.879	0.015*	3.532(1.301-9.593)	0.013	9.696	0.002*	5.644(1.430-22.277)	0.013
Menopausal Status	4.524	0.033*			4.644	0.031*		
Tumor Location	0.233	0.629			0.078	0.780		
Histologic Type	2.992	0.084			2.16	0.142		
Clinical Stage	16.067	<0.001*			19.806	<0.001*		
Clinical T Stage	1.273	0.736			6.472	0.091		
Clinical N Stage	9.238	0.010*			16.255	<0.001*		
Clinical M Stage	15.472	<0.001*	2.977(1.217-7.283)	0.017	19.46	<0.001*	3.499(0.962-12.727)	0.057
ER Status	3.902	0.048*			3.678	0.055		
PR Status	5.084	0.024*			5.357	0.021*		
HER2 Status	0.027	0.870			0.035	0.853		
Molecular Subtype	4.626	0.201			4.763	0.190		
Ki-67	4.174	0.041*			1.157	0.282		
Treatment	5.994	0.050			4.931	0.085		
SUV_max_	7.058	0.008*			2.028	0.154		
SUV_min_	5.359	0.021*	4.240(1.814-9.910)	0.001	2.598	0.107		
SUV_mean_	3.950	0.047*			1.686	0.194		
SUV_peak_	6.130	0.013*			1.685	0.194		
MTV	1.772	0.183			3.848	0.050		
TLG	2.891	0.089			3.758	0.053		
Rad-score	4.261	0.039*	2.660(1.029-6.880)	0.044	6.930	0.008*	3.627(1.171-11.241)	0.026

The clinicopathological factors and Rad-score were analyzed by the log-rank method in univariate analysis, and then characteristics with p < 0.05 were taken into the multivariate Cox regression to construct the final model. HR, hazard ratio; CI, confidence interval; ER, estrogen receptor; PR, progesterone receptor; HER2, human epidermal growth factor receptor 2; SUV, standardized uptake value; MTV, metabolic tumor volume; TLG, total lesion glycolysis; Rad-score, radiomic score. *P < 0.05.

### Model validation and assessments

**Figure 7:**
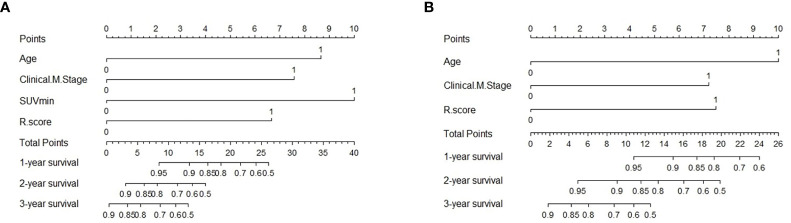
Predictive nomogram of C-R model for PFS **(A)** and OS **(B)** in the testing cohort. Summed by the points of every risk factor, the final points are located on the Total Point axis. R.score, radiomic score.

**Figure 8:**
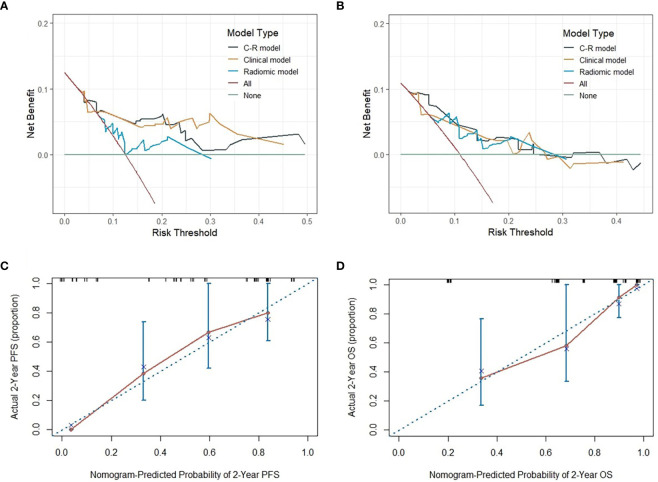
The DCA of three models for PFS and OS in the testing cohort **(A, B)**. The y-axis measures the net benefit, which is calculated by summing the benefits (true positive results) and subtracting the harms (false-positive results). The calibration curves of C-R models’ nomograms for the 2-year probability of PFS and OS **(C, D)**. The dashed line indicated a perfect match between the actual probability (y-axis) and the nomogram-predicted probability of 2-year PFS and OS (x-axis).

To evaluate the probability of 1-, 2-, and 3-year PFS and OS, we established nomograms for an integrated model that incorporated the most valuable clinical and imaging parameters (**Figure 7**). For the training cohort, the concordance index (C-index) and AUC of the C-R model were 0.786 (95% CI: 0.697 - 0.875) and 0.787 (95% CI: 0.667 - 0.880), respectively, for PFS prediction. These values were superior to those of the single clinical or radiomic models, demonstrating the good predictive accuracy of the C-R model (**Table 2**). Similar performance was observed for OS, where the C-index and AUC of the C-R model were 0.878 (95% CI: 0.816 - 0.940) and 0.789 (95% CI: 0.669 - 0.881), respectively, and were higher than those of other models. The C-R model was successfully validated in the testing set. In the validation cohort, the C-index of the C-R model was 0.816 (95% CI: 0.685 - 0.947) for predicting PFS and 0.882 (95% CI: 0.781 - 0.984) for predicting OS. Using ROC curve analysis, we found that the C-R model had a higher AUC compared with the other two models for predicting PFS (AUC = 0.830, 95% CI: 0.636 - 0.946) and OS (AUC = 0.859, 95% CI: 0.671 - 0.962) (**Table 2**). **Figure 8** presents the DCA and calibration curve of the nomogram (2-year predictive probability of the C-R model) for PFS and OS. The DCA for the PFS nomogram indicated that the net benefit of the clinical and C-R models was slightly higher compared with the radiomic model within reasonable threshold probabilities (**Figure 8A**). Regarding OS, while there was no significant difference observed among the three models, all of them provided overall net benefits in clinical application (**Figure 8B**). Both calibration curves of the nomograms for PFS and OS showed accurate discrimination between prediction and observation in the testing set (**Figures 8C, D**).

## Discussion

Recent studies have revealed various clinical endpoints among individuals with BC, and precise prediction based on non-invasive machine learning methods has become increasingly prevalent in prognostic research (29–31). These promising results have encouraged the emergence of combination models based on clinical, histological, and imaging features to better meet clinical requirements (32, 33). In this study, we established novel nomograms that reflected the underlying role of clinicopathological and radiomic biomarkers extracted from ^18^F-FDG PET/CT to estimate the outcomes of BC patients. Regarding the prediction of PFS, the C-index of the C-R model, clinical model, and radiomic model was 0.786, 0.761, and 0.613 in the training group and 0.878, 0.794, and 0.730 in the testing group, respectively. With regard to the endpoint of OS in the training test, the C-index of the three models was 0.816, 0.772, and 0.674, and it became 0.882, 0.731, and 0.706 in the testing test, respectively. These findings suggested the superior predictive performance of the combination model in both the training and validation cohorts compared to other single models.

Traditionally, clinicians have relied on TNM staging to make prognostic assessments based on physical examinations and clinical symptoms. With the emergence of PET/CT scans, these assessments have been improved. However, patients at the same stage still show varying outcomes, even when treated with similar strategies, as the stage changes. Therefore, there is an urgent clinical need for an accurate predictive method of prognosis, particularly for highly heterogeneous malignancies, such as BC. In our retrospective study, metabolic parameters of PET/CT played an invaluable role in predicting the risk of disease recurrence. Shingo Baba et al. have revealed a correlation between higher SUVs extracted from PET images and a poor prognosis of BC. On the other hand, Evangelista et al. have found that MTV and TLG are independent factors in predicting BC recurrence, while SUV_max_ demonstrates poor prognostic performance. However, it is important to note that metabolic parameters can be influenced by various physiological and technical factors. In the end, only SUV_min_ is identified as independently associated with PFS.

To comprehensively quantify tumor heterogeneity, radiomics can provide more detailed information on the tumor microenvironment beyond visual features, allowing for the reflection of multiple clinical endpoints. High-dimensional features, such as NGLDM and GLZLM, have been found to be associated with survival time in various tumor types and have been used in the construction of the Rad-score (34–36). These studies have also confirmed that Rad-score is an independent biomarker for predicting survival status. In our present study, NGLDM_Coarseness_, NGLDM_Contrast_, and GLZLM_GLNU_ were selected using LASSO regression and used to calculate the Rad-score, which was found to be a meaningful predictor of both PFS and OS. As far as we know, our study is one of the few articles that have focused on predicting BC prognosis using PET/CT imaging and histology.

In addition, age and clinical M stage were also identified as prominent predictors for both PFS and OS in our study. Specifically, our study found that patients over the age of 50 and those with distant metastases (clinical M stage: M1) and higher Rad-score were more likely to experience earlier disease progression or even death. Moreover, recent research has confirmed that visually represented nomograms based on clinicopathologic risk factors and rad-score can significantly contribute to the prediction of prognosis (37). The C-index, along with its 95% CI, DCA, and calibration curve in the testing cohort, can provide a more comprehensive assessment, including discrimination, clinical applicability, and calibration of the nomogram for the predictive model (38). Therefore, we aimed to establish an integrated model visualized by a nomogram to assess the potential prognostic value of BC patients by combining PET/CT-based radiomics with clinical features.

However, our work has some limitations that need to be acknowledged. Firstly, our limited population needs to be taken into account, although it is homogeneous in terms of histology types. Our assumptions need to be further strengthened in multicenter prospective cohorts. Secondly, previous literature has shown that radiological signatures derived from PET/CT may be influenced by the equipment and software used for image acquisition, reconstruction, and analysis (39). All patients underwent PET/CT examination with consistent scanners in our study. Thirdly, although the inter-observer agreement was repeatable (ICC > 0.75), selection bias was inevitable. Lastly, the survival study highly depended on follow-up time, and adequate interviews will be necessary to validate our results.

## Conclusion

In conclusion, we established a complex model that incorporated both clinicopathologic and radiomic factors, which could potentially serve as a biomarker for risk stratification of prognosis in patients with invasive BC. Our strategy demonstrated not only a great net benefit at a large range of threshold probabilities but also accurate discrimination in clinical applications.

## Data availability statement

The original contributions presented in the study are included in the article/supplementary material. Further inquiries can be directed to the corresponding authors.

## Ethics statement

The studies involving human participants were reviewed and approved by the First Affiliated Hospital of Soochow University. The ethics committee waived the requirement of written informed consent for participation.

## Author contributions

TJ, QL, XC, and SD conceptualized and designed the study. SG and SS performed analysis. BZ and CY interpreted the data. TJ and QL drafted the manuscript. SS and SD revised the manuscript. All authors contributed to the article and approved the submitted version.
